# Differential Homing Receptor Profiles of Lymphocytes Induced by Attenuated versus Live *Plasmodium falciparum* Sporozoites

**DOI:** 10.3390/vaccines10101768

**Published:** 2022-10-21

**Authors:** Marie Mura, Tanmaya Atre, Tatyana Savransky, Elke S. Bergmann-Leitner

**Affiliations:** 1 Immunology Core, Biologics Research & Development, WRAIR—Walter Reed Army Institute of Research, Silver Spring, MD 20910, USA; 2 Immunopathology, Microbiology and Infectious Diseases, IRBA—Institut de Recherche Biomédicale des Armées, 91220 Brétigny-sur-Orge, France; 3 Experimental Medicine, BC Children’s Hospital Research Institute, Vancouver, BC V5Z 4H4, Canada; 4 Division of Entomology, WRAIR—Walter Reed Army Institute of Research, Silver Spring, MD 20910, USA

**Keywords:** lymphocyte homing, vaccine, malaria, flow cytometry, parasite, *Plasmodium*

## Abstract

The onset of an adaptive immune response provides the signals required for differentiation of antigen-specific lymphocytes into effector cells and imprinting of these cells for re-circulation to the most appropriate anatomical site (i.e., homing). Lymphocyte homing is governed by the expression of tissue-specific lymphocyte homing receptors that bind to unique tissue-specific ligands on endothelial cells. In this study, a whole-parasite malaria vaccine (radiation-attenuated sporozoites (RAS)) was used as a model system to establish homing receptor signatures induced by the parasite delivered through mosquito bite to provide a benchmark of desirable homing receptors for malaria vaccine developers. This immunization regimen resulted in the priming of antigen-specific B cells and CD8^+^ T cells for homing primarily to the skin and T/B cell compartments of secondary lymphoid organs. Infection with live sporozoites, however, triggers the upregulation of homing receptor for the liver and the skin, demonstrating that there is a difference in the signal provided by attenuated vs. live sporozoites. This is the first report on imprinting of homing routes by *Plasmodium* sporozoites and, surprisingly, it also points to additional, yet to be identified, signals provided by live parasites that prime lymphocytes for homing to the liver. The data also demonstrate the utility of this method for assessing the potential of vaccine formulations to direct antigen-specific lymphocytes to the most relevant anatomical site, thus potentially impacting vaccine efficacy.

## 1. Introduction

Lymphocyte homing is a highly regulated trafficking process responsible for homeostasis of leukocyte subsets in tissues and organs. It dispatches the immunological repertoire in a targeted manner, and thus leads to the preferential presence of specific immune cell subsets in a specialized microenvironment, presumably the sites where a pathogen will most likely be encountered. Lymphocyte homing is controlled by their expression of “homing receptors”, including selectins, integrins, and chemokine receptors, and their respective ligands on vasculature. Three distinct steps involved in the process have been described: rolling of leukocytes along endothelial cells (EC), firm adhesion of leukocytes to EC, and diapedesis. The specific pattern of homing receptors expressed on a cell determines its preferred place and circulation route through the body, but this process is dynamic and changes after an initial antigen encounter. As naïve lymphocytes encounter their specific antigen for the first time, they are primed to preferentially home to this anatomically site throughout their life (a concept referred to as “There is no place like home”) [1]. Circulation of naïve T cells into secondary lymphoid organs (SLO) is based on the expression of L-selectin (CD62L) and chemokine receptor CCR7. These two homing receptors are downregulated upon differentiation of the lymphocytes into effector cells, and other receptors are upregulated to enable entry into non-lymphoid tissues, such as CCR9 that directs cells to the gut, or the skin-specific CCR10 [2]. Within memory T cells, there are differences in homing between effector memory T cells (Tem) and central memory T cells (Tcm): the first circulates through non-lymphoid tissues whereas the latter homes to secondary lymphoid organs [3,4]. The third type of memory T cells are tissue-resident memory cells (Trm) that are maintained by renewal through differentiation of effector T cells within tissues [5]. Trm are an important subset of non-circulating, highly specialized T cells that are found at multiple barrier and mucosal sites, which enables them to rapidly react to a specific antigen. Trm adapt to specific anatomical sites by changes in their transcriptomics profile and epigenetic modifications [6]. There is growing evidence of their importance in protection against several diseases, such as liver Trm in the case of malaria [7,8] or pulmonary Trm in the case of influenza [9]. The relevance of Trm has also recently been demonstrated in a study revealing age-dependent, quantitative and qualitative differences in Trm induced by oral immunization with the Ty21a *Salmonella typhi* vaccine [10].

How immunization route and vaccine platforms may impact lymphocyte homing and imprinting of the immune response is of great interest because it would help with the design of more effective vaccines that direct the immune response to tissues primarily affected by the pathogen. Immune imprinting is a phenomenon whereby the initial exposure to an antigen effectively primes lymphocyte memory but limits the development of a different response pattern that might be necessary to protect against a variant or another strain of the same pathogen after a secondary encounter of the antigen. It has been shown for viruses such as influenza that the mode of initial exposure to a virus or a vaccine affects both, the strength of the response and the breadth of the imprint [11,12]. The expression of a particular set of homing receptors can help to determine the eventual destination of an activated T cell, and how local tissue-immunity is imprinted. For instance, immunization through mucosal or cutaneous routes leads to the upregulation of gut- and skin-homing receptors, respectively [5]. More specifically, it has been shown that tissue-associated dendritic cells appear to be capable of imprinting the tropism of a T cell during the priming phase. 

The impact of vaccination routes on bacterial vaccines is well known, especially in the context of mucosal homing. Oral and systemic typhoid immunization result in differences in lymphocyte homing to tissues, underlying the importance of the site of Ag encounter [13,14]. Such observations support the concept that lymphocytes are “imprinted” in lymph nodes to express specific homing receptors and chemokine receptors that allow them to migrate to the targeted tissue. Furthermore, the preferential migration of the T cells to sun-exposed cutaneous sites suggests that inflammation plays a critical role in this migration [15]. Natural infection with *Streptococcus pneumoniae* induces greater mucosal localization for immune response than systemic vaccination [16]. Finally, *Shigella* oral vaccine induces plasma blast subsets with the potential to home to the gut and other secondary lymphoid organs [17]. Together, these data strongly associate the level of the mucosal immune response to the route of immunization. Less is known, however, about viral vaccines. The viral vector Ad5 used in an HIV trial showed that pre-existing adenoviral immunity leads to a preferential expansion of HIV-susceptible activated CD4 T cells that home to mucosal tissues [18]. SARS-CoV-2-specific T cells from convalescent mRNA-vaccinees differed strikingly from those isolated from infection-naïve mRNA-vaccinees, with phenotypic features suggesting a superior long-term persistence and ability to home to the respiratory tract after natural infection [19]. Intramuscular or intradermal influenza vaccination is also relatively inefficient at generating plasmablasts capable of immediate trafficking to mucosal sites, as compared with natural infection [20]; however, the vast majority of vaccines are delivered via those routes.

Therefore, monitoring the expression of homing receptors on antigen-specific lymphocytes provides a tool to define to which tissue these cells will migrate, and thus having a higher likelihood of providing protective efficacy. Homing increases the frequency of either circulating effector/memory cells or tissue-resident lymphocytes at a site where the initially encountered pathogen will most likely re-infect the host. A major challenge for vaccines is to ensure that lymphocytes are primed for the correct homing route, which may not occur when the vaccine is delivered through a route that differs from that the pathogen uses to enter the host. 

Currently, virtually nothing is known about homing patterns induced by the malaria parasite or the different vaccine candidates in development. Despite the WHO’s recommendation regarding the use of the RTS, S sub-unit vaccine in endemic areas, there is still an urgent need for the development of more effective second generation vaccines. The most promising contemporary approach is the use of an attenuated whole-parasite vaccine that is based on either chemically, radiation or genetically attenuated sporozoites (SPZ). The radiation-attenuated vaccine *Pf*SPZ induces differences in the systemic immune response depending on the route of delivery, with a clear impact of the route of immunization on vaccine efficacy. Animal studies (nonhuman primates, mice) demonstrated that intravenous immunization was critical for inducing a high frequency of *Pf*SPZ-specific CD8^+^ IFN-γ-producing T cells in the liver and conferring protection, as compared with subcutaneous and intramuscular routes of immunization [21]. What is unknown, however, is the homing pattern induced by sporozoites. It has been shown that effector immune mechanisms that mediate sterile protection against *Plasmodium* arise in the skin, the skin draining lymph nodes, and the liver [22,23,24,25]. The importance of T cell priming in skin draining lymph nodes to generate T cells that circulate to the liver has been demonstrated in murine studies [26]. CD11c^+^ dendritic cell subsets in the skin draining lymph nodes are critical for priming *Plasmodium*-specific CD8+ T cells. Moreover, studies in non-human primates suggest that the *Pf*SPZ vaccine confers durable protection against malaria through long-lived tissue-resident *Pf*-specific, interferon-γ-producing CD8 T cells [27]. 

In this study, we sought to define lymphocytes homing receptor profiles induced by immunization via mosquito-delivered, radiation-attenuated *P. falciparum* sporozoites (IMRAS). We aim to provide a benchmark for future vaccine design in regard to “desirable” homing receptor patterns, and to determine whether there are differences between live vs. attenuated sporozoites.

## 2. Materials and Methods

### 2.1. Study Design

Samples for this study, i.e., peripheral blood mononuclear cells (PBMCs), were collected under a clinical protocol (www.clinicaltrials.gov trial ID NCT01994525, accessed on 12 January 2022) from an open-label clinical study for safety and identification of biomarkers of protection in two cohorts of healthy malaria-naïve adults, who had gone through five immunization sessions involving bites from *Anopheles stephensi* mosquitoes that were either infected with PfRAS (true-immunization, n = 21) or non-infected (mock-immunization, n = 5). Leukapheresis samples were available from four time points: pre-immune (T0) as a reference sample for each volunteer; post-immune (T1), after the third immunization (pre-CHMI), to establish a signature of immunization; day 5–6 post-CHMI (T2) as an early time point after infectious SPZ inoculation via mosquito bites; and 3–4 months post-CHMI (T3) to determine evidence of editing of the immune response by the CHMI.

### 2.2. Flow Cytometric Analysis

Cryopreserved PBMCs from the four time points were cultured either in media alone (negative control) or stimulated with sporozoites (SPZ) isolated from salivary glands of *Anopheles stephensi* infected with NF54 *Pf* (15,000 lysed SPZ/per 1 × 10^6^ PBMCs). Cells were cultured for 24 h (37 °C, 5% CO_2_) in complete medium (RPMI-1640 (Life Technologies, Waltham, MA, USA) containing 10% human serum (Gemini Bio-Products, West Sacramento, CA, USA)) at a concentration of 1 × 10^7^ cells/mL. Following antigen stimulation, PBMCs were washed and aliquoted to be stained with the following antibody mixtures (Supplementary Table S1 for clone information, dilution, and vendor): anti-human CD3-Vioblue, anti-human CD4-FITC, anti-human CD8-PerCP-Vio700, anti-human CD19-VioGreen, anti-CD69-APC-Vio 770, viability dye (LIVE DEAD^TM^ fixable blue viability dye, Invitrogen)), and antibodies of *Panel 1* (anti-human CD186-PE, anti-human CD185-PE-Vio770, anti-human CD161-APC), or Panel 2 (anti human CCR10-PE, anti-human CD183-PE-Cy7, anti-human CD196-APC), or Panel 3 (anti-human CCR9-PE, anti-human CD197-PE-Cy7, anti-human CD103-APC), for 15 min at 4 °C in FACS solution (0.5% human serum and 0.1% sodium azide in PBS). Cells were washed in FACS solution and acquired on a BD LSR Fortessa. Cell viability of thawed PBMCs was >92% as measured by a Luna-FL™ Dual Fluorescence cell counter (fluorescence protocol with acridine orange/propidium iodide (AO/PI) to determine cell viability). Viability of the cells after over-night stimulation and staining was >84%. Lymphocytes were first gated based on Forward- (FSC) vs. Sideward Scatter (SSC), then single cells, followed by viability, and lineage markers (Supplementary Figure S1). CD69 expression was used to gate antigen-specific cells before assessing the expression of the different homing receptors. CD69 is an early-activated marker with rapid expression on a broad range of immune cells that makes it amenable for the early detection of lymphocyte activation. Its rapid expression (<2 h post-activation) on a broad range of immune cells (T cells, B cells, NK cells) makes it suitable for the early detection of T cell activation and for subset activation analyses [28]. Flow cytometric data were analyzed using FlowJo V10 (Treestar, Ashland, OR, USA). Raw data were used for the statistical analyses, rather than data after subtracting the values from media-stimulated (background) controls.

### 2.3. Statistical Analysis

Univariate analysis: To determine immunization-induced changes in the expression of homing receptors, we carried out a univariate analysis for each marker, comparing pre-immune (T0) vs. post-immune (T1) responses, and post-immune (T1) vs. post-CHMI (T2, T3). If normally distributed, as determined by Shapiro–Wilks tests, paired Student’s *t*-tests were used to calculate statistical significance. If not normally distributed, the Wilcoxon signed-rank test was applied. After calculating *p*-values for immune parameters in the datasets, a correction for multiple comparisons was made (resulting in a corrected *p*-value) using the Benjamini–Hochberg correction. Immune measures in which comparison to the pre-immune data showed a significant difference at *p* < 0.05 and a false discovery rate of q < 0.20 were selected for multivariate analyses. 

Multivariate analysis correlation matrices for the dataset were generated by calculating the Spearman correlation coefficient between all immune measures. Spearman’s ρ statistic was used to calculate *p*-values for each correlation estimate. Only correlation coefficients with *p* < 0.05 were retained for further analysis to ensure that only high-confidence correlations were used in subsequent analyses; all others were set to ‘0’. Hierarchical clustering (R package *hclust* function) was used to group correlated immune measures and to define immune clusters based on a cutoff criterion of having a correlation coefficient of at least 0.40, using the R package *cutree* function.

## 3. Results

We used samples from a whole-parasite vaccine to determine whether immunization via the natural route, i.e., mosquito bite primes antigen-specific lymphocytes for a specific homing route. For several decades, various aspects of the irradiated sporozoite vaccine (RAS) have been investigated since this vaccine is highly protective, thus providing benchmarks for the development of efficacious malaria vaccines that are easier to produce and administer. The vaccination route has been one of the most studied parameters as only delivery by mosquito bite itself or intravenous injections have resulted in complete sterile protection [29,30]. We screened the literature to identify homing receptors responsible for mediating migration of lymphocytes into tissues that are relevant during a Plasmodium infection (i.e., skin, liver) and designed flow cytometric panels to monitor changes in expression of nine different receptors on peripheral blood mononuclear cells (Table 1, Supplementary Table S1). 

### 3.1. Immunization and Challenge with Live Sporozoites Induce Significant Changes in the Frequency of SPZ-Specific B Cells with a Unique Homing Receptor Profile

**Figure 1:**
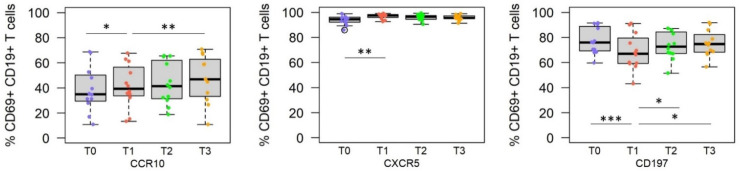
Homing receptor profiles of SPZ-specific B cells at different time points after vaccination and CHMI. Statistical analysis revealed that the frequency of CD69^+^ B cells expressing the skin-specific marker CCR10 (left panel), B cell compartment-specific CXCR5 (middle panel), and T cell compartment -specific CD197 (right panel) significantly change in the course of the immunization and after parasite challenge (infection) ((T0 = pre-immune, T1 = 2 weeks after the third immunization, T2 = 5–6 days after CHMI, T3 = 3–4 months after CHMI). Asterisk indicates statistical significance (* *p* < 0.05, ** *p* < 0.01, *** *p* < 0.001)).

Longitudinally collected peripheral blood mononuclear cells from individuals immunized with the irradiated SPZ vaccine (RAS) were thawed and stimulated with Plasmodium falciparum sporozoites or cultured in complete medium (negative control) overnight before the analysis. This overnight stimulation led to the upregulation of the activation marker CD69 that serves as a surrogate marker for antigen-specificity [38]. Statistical analysis revealed a clear difference in the homing receptor profiles of sporozoite-specific B cells at the different time points: pre-immune (T0) vs. post-immune (T1), pre-challenge (T2) vs. post-challenge (T3) (Figure 1). Immunization led to a significantly higher frequency of sporozoite-specific B cells expressing the homing receptor CXCR5 (*p* = 0.003) that allows entry into the B cell compartments of secondary lymphoid organs, and CCR10, a homing receptor for the skin (*p* = 0.04). The frequency of SPZ-specific B cells expressing CD197, a homing receptor for T cell compartments (*p* < 0.001), was significantly reduced. Samples collected after controlled human malaria infection (CHMI) contained significantly higher frequencies of SPZ-specific B cells expression of CCR10 (T1 vs. T3, *p* = 0.008/T0 vs. T3, *p* = 0.006), a homing receptor for the skin, whereas the frequency of B cells expressing CD197 returned to its pre-immune state (T1 vs. T3, *p* = 0.013/T0 vs. T3, *p* = ns). 

### 3.2. Challenge with Live Sporozoites, but Not Immunization, Induces Significant Changes in the Homing Receptor Profiles of SPZ-Specific CD4^+^ T Cells 

**Figure 2:**
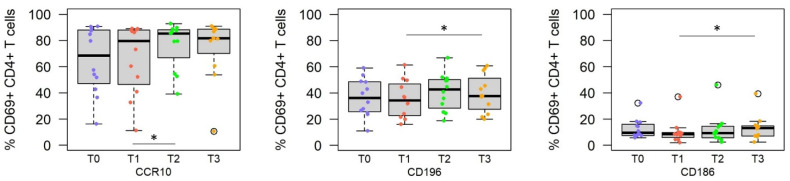
Homing receptor profile of SPZ-specific CD4^+^ T cells at different time points after vaccination and CHMI. Statistical analysis revealed that the frequency of CD69^+^ CD4^+^ T cells expressing the skin-specific marker CCR10 (left panel), CD196 (middle panel), and liver-specific CD186 (right panel) was significantly altered when testing samples obtained at q different time points (T0 = pre-immune, T1 = 2 weeks after the third immunization, T2 = 5–6 days after CHMI, T3 = 3–4 months after CHMI). Asterisk indicates statistical significance (* *p* < 0.05).

Immunization with the RAS vaccine did not induce significant changes in the expression of homing receptors on SPZ-specific CD4^+^ T cells (Figure 2). In contrast, challenge of the vaccinees with live SPZ significantly increased the frequency of CD186^+^ CD4^+^ T cells (homing receptor for liver, comparing T1 vs. T3, *p* = 0.012) and the skin homing receptors CD196 and CCR10-expressing CD4+ T cells, comparing T1 vs. T2, T3 (*p* = 0.016 and *p* = 0.010, respectively).

### 3.3. RAS Vaccine and Live SPZ Challenge Induce a Complex Profile of Homing Receptors on SPZ-Specific CD8^+^ T Cells

**Figure 3:**
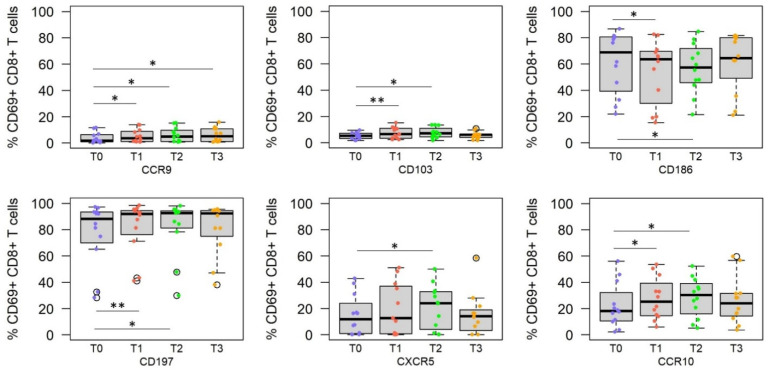
Homing receptor profile of SPZ-specific CD8^+^ T cells at different time points after vaccination and CHMI. Statistical analysis revealed that the frequency of CD69^+^ CD8^+^ T cells expressing CCR9 (top left), CD103 (top middle), CD186 (top right), CD197 (bottom left), CXCR5 (bottom middle), and CCR10 (bottom right) was significantly altered when testing at different time points (T0 = pre-immune, T1 = 2 weeks after the third immunization, T2 = 5–6 days after CHMI, T3 = 3–4 months after CHMI). Asterisk indicates statistical significance (* *p* < 0.05, ** *p* < 0.01).

Defining the homing receptor profile of longitudinally collected peripheral blood, SPZ-specific CD8^+^ T cells revealed upregulation of a variety of homing receptors resulting in a much more complex pattern compared with CD4^+^ T cells and B cells (Figure 3). Immunization with the RAS vaccine induced a significant increase in the frequency of peripheral SPZ-specific CD8^+^ T cells expressing the homing receptors CCR9 (*p* = 0.016), CD103 (*p* = 0.007), CCR10 (*p* = 0.022), CD197 (*p* = 0.004), but not CD186 (*p* = 0.031). Challenge with live SPZ increased the frequency of cells expressing CCR9 (*p* = 0.016), CD103 (*p* = 0.014), CCR10 (*p* = 0.032), CD197 (*p* = 0.012), and CXCR5 (*p* = 0.020) in peripheral blood. Interestingly, while vaccination (T1) and the early time point post-challenge (T2) result in a significant reduction in the frequency of CD186^+^ CD69^+^CD8^+^ T cells and an increase in the frequency of CCR10^hi^ CD69^+^CD8^+^ T cells, the frequency of these cell populations are restored in peripheral blood 3 to 4 months post-challenge (T3).

In summary, exposure to attenuated (T1) and live sporozoites (T2, T3) results in a distinct homing receptor profile of antigen-specific B and T cell subsets (Table 2). 

### 3.4. Homing Receptor Profiles

A correlation analysis was performed on post-vaccination and post-challenge samples to define homing receptor signatures and the relationships between the different markers (Supplementary Figure S2). We noticed that the expression of secondary lymphoid organ markers (i.e., CXCR5, CD197) correlated positively with the expression of skin and gastro-intestinal homing markers (CCR10 and CCR9, respectively) expressed on CD8^+^ T cells. These markers correlated negatively with markers of homing to the liver (CD186), inflamed tissue (i.e., CD161, CD196), and epithelial tissues (CD103). 

**Figure 4:**
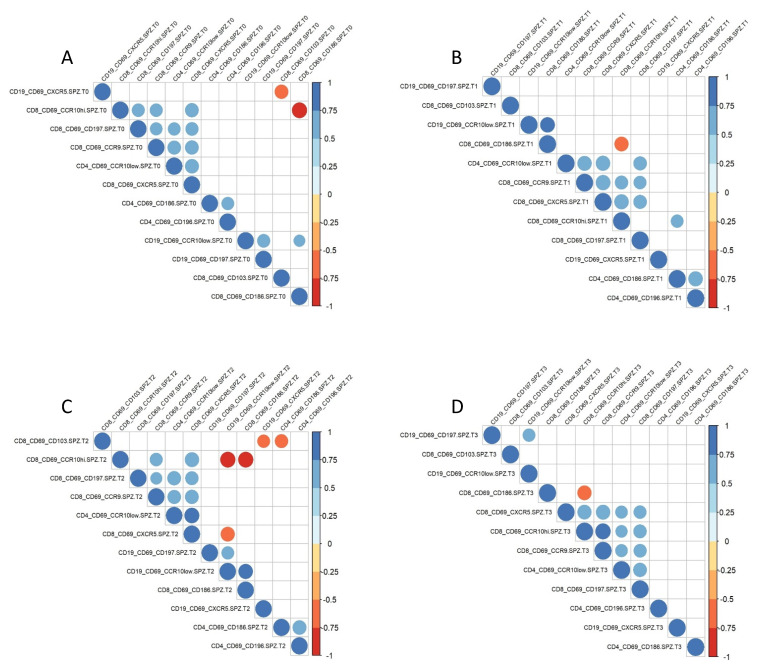
Homing receptor profiles induced by RAS vaccination. The relationship between expressed homing receptors on SPZ-specific lymphocytes was investigated by generating a correlation matrix including only homing receptors with significant altered expression after immunization or challenge. (**A**) Correlation matrix of these 12 parameters before immunization (T0); (**B**) after immunization (T1); (**C**) 5–6 days after CHMI (T2); (**D**) 3–4 months after CHMI (T3). Only significant correlations (*p* < 0.05) are shown. The color and size of the dots (scale next to graph) indicate the degree of correlation between the different parameters (small to large indicating low to high correlation).

We next established the profiles of homing receptor expression on antigen-specific (CD69^+^) CD19^+^ B cells, CD4^+^ and CD8^+^ T cells at the different time points by generating correlation matrices using only markers that were significantly different from baseline (Figure 4).

At baseline (T0, Figure 4A), there were two significant negative correlations, i.e., (i) the frequency of antigen-specific B cells expressing CXCR5 vs. the frequency of antigen-specific CD8^+^ T cells expressing CD103), (ii) the frequency of antigen-specific CD8^+^ CCR10^+^ T cells vs. CD8^+^ CD186^+^ T cells. There were several positive correlations within the antigen-specific lymphocyte populations such as the frequency of CD8^+^ T cells homing to the skin and CD8^+^ T cells homing either to intestinal tissues (CCR9) or secondary lymphatic organs (CD197). 

At the post-immune time point (T1, Figure 4B), the frequency of skin-homing CCR10^+^ antigen-specific CD8^+^ T cells correlated negatively with CD186^+^ CD8^+^ T cells homing to the liver. The positive correlations were all linked to the CCR10^+^ CD8^+^ T cell population. The frequencies of antigen-specific B cells and CD4^+^T cells likely homing to the liver (CD186) correlated with cells likely homing to the skin and inflamed tissues (CD196). 

We generated correlation matrices also for the post-challenge time points to determine whether exposure to live *Pf*SPZ induced a different profile compared with radiation-attenuated SPZ: shortly after challenge (T2, Figure 4C). Within the antigen-specific populations, the frequencies of CD8^+^ T cells expressing CCR10^+^ or CXCR5^+^ correlated negatively with B cells expressing CCR10. We also observed a negative correlation between the frequency of CD103^+^ CD8^+^ T cells with CXCR5^+^ B cells. Most of the positive correlations were observed within the antigen-specific CD8^+^ T cells bound to various tissues. In addition, the frequencies of B cells homing to secondary lymphatic organs (CD197) correlated with those B cells destined for the skin (CCR10). 

The negative correlation between antigen-specific CD8^+^ CCR10^+^ T cells and antigen CD69^+^ CD8^+^ CD186^+^ T cells could still be detected at the final time point (3-4 months post-challenge, Figure 4D). All other significant correlations were found within the antigen-specific CD8^+^ T cell population expressing CXCR5.

We conclude that the homing receptor profiles of antigen-specific B and T cells after vaccination likely mediates more homing of these cells into the skin, while the injection of infectious/live sporozoites promotes homing of SPZ-specific lymphocytes to the liver.

## 4. Discussion

The objective of this study was to determine whether evaluating the expression pattern of homing receptors on vaccine-induced lymphocytes could provide valuable insights into how to improve vaccine design. Here, we specifically focused on the impact of vaccine formulation; however, the approach can also be applied to optimizing vaccination route and other parameters. As our model vaccine, we selected a whole-parasite vaccine since previous reports have demonstrated that only intravenous administration of purified, irradiated sporozoites (DVI) [30,39] was able to reproduce the high level of protective efficacy that is achieved by immunization via irradiated mosquito bites (IMRAS) [29,40]. To achieve full protective efficacy, the immunization regimen for IMRAS typically requires more than 1000 bites of irradiated *Pf*SPZ-infected mosquitoes [41]. The samples used for this study were derived from individuals who had been immunized by receiving approximately 200 mosquito bites per session in the course of five immunization sessions [29], which explains the tropism for skin and lymph nodes by vaccine-induced lymphocytes. Mouse studies have shown that roughly 100 *P. berghei* SPZ are delivered into skin by each bite [42]. Assuming a similar efficiency of *Pf*SPZ-infected mosquitoes, we conclude that each immunization session would deliver approximately 2000 SPZ into the skin. 

We identified homing receptors (chemokine receptors, αEβ7-integrin and C-type lectin) associated with homing into tissues relevant for malaria (i.e., skin, liver) through literature searches. Next, we evaluated longitudinal samples from individuals immunized with IMRAS and then challenged with infectious SPZ. We measured the expression of homing receptor expression on antigen-specific B and T cell subsets by flow cytometry to establish vaccine-induced homing receptor profiles: immunization with the RAS vaccine delivered by mosquito bite (i.e., IMRAS) resulted in the priming of antigen-specific B cells and CD8^+^ T cells for homing primarily to the skin and T/B cell compartments of secondary lymphoid organs. Challenge (i.e., infection) with live sporozoites, however, triggered the upregulation of homing receptor for the liver and the skin demonstrating that attenuated vs. live SPZ deliver different signals.

IMRAS immunization induced the upregulation of CCR10 on SPZ-specific B cell, a chemokine receptor that mediates lymphocyte homing into the skin. Similarly, we observed expression of CXCR5 that is associated with homing into germinal centers of lymph nodes while CD197 required for lymphocyte homing into the T cell compartments of the lymph nodes was downregulated. No significant changes in the expression profile of homing receptors were observed on SPZ-specific CD4^+^ T cells. In contrast, the homing receptor profile of SPZ-specific CD8^+^ T cells was greatly altered by higher expression of skin (CCR10) and gut (CCR9) homing receptors, as well as the αEβ7-integrin CD103. Interestingly, CD186, a homing receptor for the liver [33] was downregulated after vaccination, but upregulated after challenge with live/infectious SPZ

CD103 contributes greatly to Trm cell adhesion in the epidermis and influences local retention. Experiments in knock down mice revealed that CXCR6 expression was required for the formation and maintenance of Trm in the skin, and CCR10 expression was involved in their retention and survival [43]. Although not critical for epidermal Trm cell migration, the lack of expression of these two homing receptors impaired the formation and survival of skin-resident memory Trm. Together, the upregulation of CD103 and CCR10 are consistent with the homing of effector lymphocytes to the skin after RAS immunization via mosquito bites. 

Exposure to infectious/live sporozoites (CHMI) unexpectedly modified the homing receptor profile observed after RAS vaccination (Table 2). At the early time point after the infectious challenge (5–6 days post-inoculation), B cells expressed significantly higher CD197 levels than after immunization, and CD8^+^ T cells expressed higher levels of CXCR5, suggesting modified homing to secondary lymphoid organs. In contrast, the expression levels of CD186, CXCR5, CCR9, CCR10 and CD103 were unaffected by the infection. At the last time point (3–4 months post-CHMI), CD4^+^ and CD8^+^ T cells expressed higher CD186 levels than after immunization. B cells continued to display a high expression level of CCR10 at this time point. The upregulation of CD186 expression after CHMI may be explained by the fact that radiation-attenuated SPZ stop developing at the early liver stage with less opportunity to stimulate immune cells in the liver compared with infectious SPZ. Indeed, infection causes extensive damage in the liver and induces a different profile of pathogen-associated molecular patterns and/or danger-associated molecular patterns, including vita-PAMPs [44]. Studies in mice have shown that CD8α^+^ dendritic cells (DC), which are responsible for the activation and induction of malaria-specific CD8^+^ T cells, accumulated at higher rates in the liver after infection than after RAS-immunization. The different effect on DC may be associated with chemokines and type-I interferon response [45]. However, the difference may also reflect the loss of CD186 expressing T cells in the blood when the majority of vaccine-induced antigen-specific T cells had already been directed to the tissues and are no longer in circulation. CD186 is a key retention marker in the liver required for residence [46,47,48] and is expressed by CD8^+^ Trm but also CD4^+^ Trm [49,50]. 

Our study revealed that attenuated and infectious/live SPZ delivered by the same route, i.e., mosquito bite, induce different homing receptor profiles. The impact of the administration route has been reported in the process of advancing the radiation-attenuated sporozoite vaccine (*Pf*SPZ vaccine) [51]. The *Pf*SPZ vaccine consists of highly purified, radiation-attenuated sporozoites. Apart from the challenge to safely cryopreserve SPZ while maintaining their ability to induce protective immunity, other parameters that had to be determined in the process of product development included dosing, scheduling, and the administration route of injectable SPZ. These studies determined that delivery of the *Pf*SPZ vaccine via the subcutaneous, intradermal, or intramuscular route failed to result in significant protection, independently of parasite liver loads [39]. Ultimately, this vaccine achieved high protective efficacy when delivered intravenously (direct venous inoculation, DVI) [30]. We speculate, based on the data from the current study, that DVI results in the priming of antigen-specific B cells and T cells that express receptors predisposing them for homing to the liver and, therefore, a comparison between the classic IMRAS and *Pf*SPZ delivered by DVI may assist in further optimizing these whole-parasite vaccines.

Our data demonstrate changes in the homing profile of antigen-specific cells as a result of either vaccination or encounter with the live parasite. When conducting clinical trials, the most commonly accessible compartment is peripheral blood and studies are conducted under the assumption that there are corresponding circulating counterparts to tissue-resident populations with PBMCs representing the proverbial “tip of the iceberg” of an individual’s total leukocyte population. Thus, the objective of analyzing the homing receptor profile is not to report changes in the frequency of specific populations, but rather to identify changes in the expression profile of homing receptors on antigen-specific lymphocytes. 

We conclude that the screening of homing receptor profiles should be conducted routinely when profiling adjuvant and vaccine platforms since different types of adjuvants, vaccine modalities, and vaccine formulation can be expected to differentially affect homing receptor expression. The panel of assessed homing receptors in this study may be adapted when evaluating vaccines targeting other pathogens. Understanding the impact on homing receptor profiles could help with rational adjuvant/vaccine platform selection, and thus, prioritize and accelerate vaccine development. The current COVID-19 pandemic triggered vaccine development at unprecedented speed. While these vaccines had a dramatic impact on morbidity and mortality caused by the virus, they are much less effective at preventing infection, especially infection with emerging viral variants, and transmission. The induction of protective mucosal immunity, presumably required for protection against infection and transmission, by SARS-CoV-2 vaccines (mRNA and Adenovirus-vectored) detected in saliva 2–4 weeks post-vaccination was transient and significantly reduced after 3 months [52]. Vaccines delivered via the intramuscular or subcutaneous route may induce only weak and transient mucosal immunity with few exemptions such as live-attenuated vaccines including the measles vaccine that induces a strong mucosal response in the upper and lower airway after parenteral delivery (s.c.) [53]. In addition, certain adjuvants may mediate mucosal imprinting, such as the non-toxic double-mutant heat-labile toxin (dmLT) from enterotoxigenic *Escherichia coli* that triggers antigen-specific CD4^+^ T cells that upregulate the gut-homing integrin α4β7 [54]. Knowing the target tissue of an appropriate immune response against a pathogen and the homing receptor profile induced by different technologies may help design more efficient vaccines. 

## 5. Conclusions

The current study demonstrates the utility of establishing homing receptor profiles of antigen-specific lymphocytes as an integral part of vaccine evaluations. Identifying differences in the homing routes of pathogen-specific effector cells will be critical for the identification of optimal vaccine formulations. Insights into how adjuvants and vaccine platforms imprint different lymphocyte recirculation and homing patters will not only expand our knowledge of human immune responses in vivo, but get us closer to rationally designed vaccines that target effector lymphocytes to their intended target tissue for maximum efficacy

## Figures and Tables

**Table 1:** Flow cytometric panel for the identification of homing receptors expressed by antigen-specific lymphocyte population.

Marker	Receptor Type	Ligand	Function/Target Tissue
CD103 (ITGAE)	Integrin	E-cadherin	Entry into epithelial tissues including liver, skin [31]
CD161 (KLRB1)	C-type lectin	LLT-1	Entry into inflamed tissue, liver (IL-17 driven) [32]
CD183 (CXCR3)	Chemokine receptor	CXCL9, CXCL10, CXCL11	Entry into inflamed tissue, liver [33]
CD185 (CXCR5)	Chemokine receptor	CXCL13	Entry into lymph nodes, direction towards B cell-compartment (follicles/germinal centers) [34]
CD186 (CXCR6)	Chemokine receptor	CXCL16	Entry into inflamed tissue, liver [33]
CD196 (CCR6)	Chemokine receptor	CCL20	Entry into inflamed tissue, Th17, skin [35]
CD197(CCR7)	Chemokine receptor	CCL19, CCL21	Entry into secondary lymphoid organs [33,36], direction towards T cell compartments [34]
CDw199 (CCR9)	Chemokine receptor	CCL25	Entry into intestinal tissues [37]
CCR10	Chemokine receptor	CCL27, CCL28	Entry into Skin [35]

**Table 2:** Summary of significant changes in homing receptor expression.

Comparison	Changes	B Cells	CD4^+^ T Cells	CD8^+^ T Cells
T0 vs. T1 i.e., Pre- vs. Post-Immune	Upregulation	CXCR5 CCR10		CCR10 CCR9 CD103 CD197 CXCR5
	Downregulation	CD197		CD186
T1 vs. T2/T3 i.e., Immune vs. post-challenge	Upregulation	CCR10 CD197	CD186 CD196	CD186

## Data Availability

All data for this study are contained within the manuscript and the Supplementary Materials. The scripts for running the analyses are deposited and are publicly available at https://github.com/BHSAI/IMRAS (accessed on 14 August 2022).

## Supplementary Materials

The following are available online at https://www.mdpi.com/article/10.3390/vaccines10101768/s1, Figure S1: Flow cytometric gating strategy and representative plots with isotype controls. Figure S2: heatmap of homing receptor profiles induced by RAS vaccination. Table S1: Antibodies used for homing receptor analysis.
