# Supplementary material for: Differential Homing Receptor Profiles of Lymphocytes Induced by Attenuated versus Live Plasmodium falciparum Sporozoites
Source: Vaccines (Basel). 2022 Oct 21;10(10):1768. doi: 10.3390/vaccines10101768 (PMC9611729; doi:10.3390/vaccines10101768)
Supplement: Supplementary file 1 [file vaccines-10-01768-s001.zip › vaccines-1936713-supplementary.pdf]

Supplementary Table S1: Antibodies used for defining homing receptor profiles. Antibodies were separated into three separate staining panels (as assigned to specific panel)

Panel	Antibody	Fluorochrome	Source	Catalog number	Clone	Dilution
All	Viability					1:250
All	CD4	FITC	Miltenyi	130-113-253	M-T466	1:50
All	CD3	Vioblue	Miltenyi	130-114-574	BW264/56	1:50
All	CD8	PerCP Vio 700	Miltenyi	130-113-161	BW135/80	1:50
All	CD19	VioGreen	Miltenyi	130-113-378	LT20	1:50
All	CD69*	APC Vio 770	Miltenyi	130-112-616	REA824	1:200
1	CD186*	PE	Miltenyi	130-123-556	REA458	1:100
1	CD185*	PE-Vio770	Miltenyi	130-098-422	REA103	1:300
1	CD161	APC	Biolegend	BL 339912	HP-3G10	1:300
2	CCR10	PE	Biolegend	341504	6588-5	1:100
2	CD183	PE Cy7	Biolegend	353720	G025H7	1:200
2	CD196	APC	Biolegend	353413	G034E3	1:50
3	CCR9	PE	R&D System	FAB179P-100	112509	1:50
3	CD197	PE Cy7	Biolegend	BL 353226	G043H7	1:300
3	CD103	APC	Beckman Coulter	BC B06204	2G5	1:100

* indicates recombinant engineered antibodies (REA) clones

Supplementary Figure S1

Gating strategy

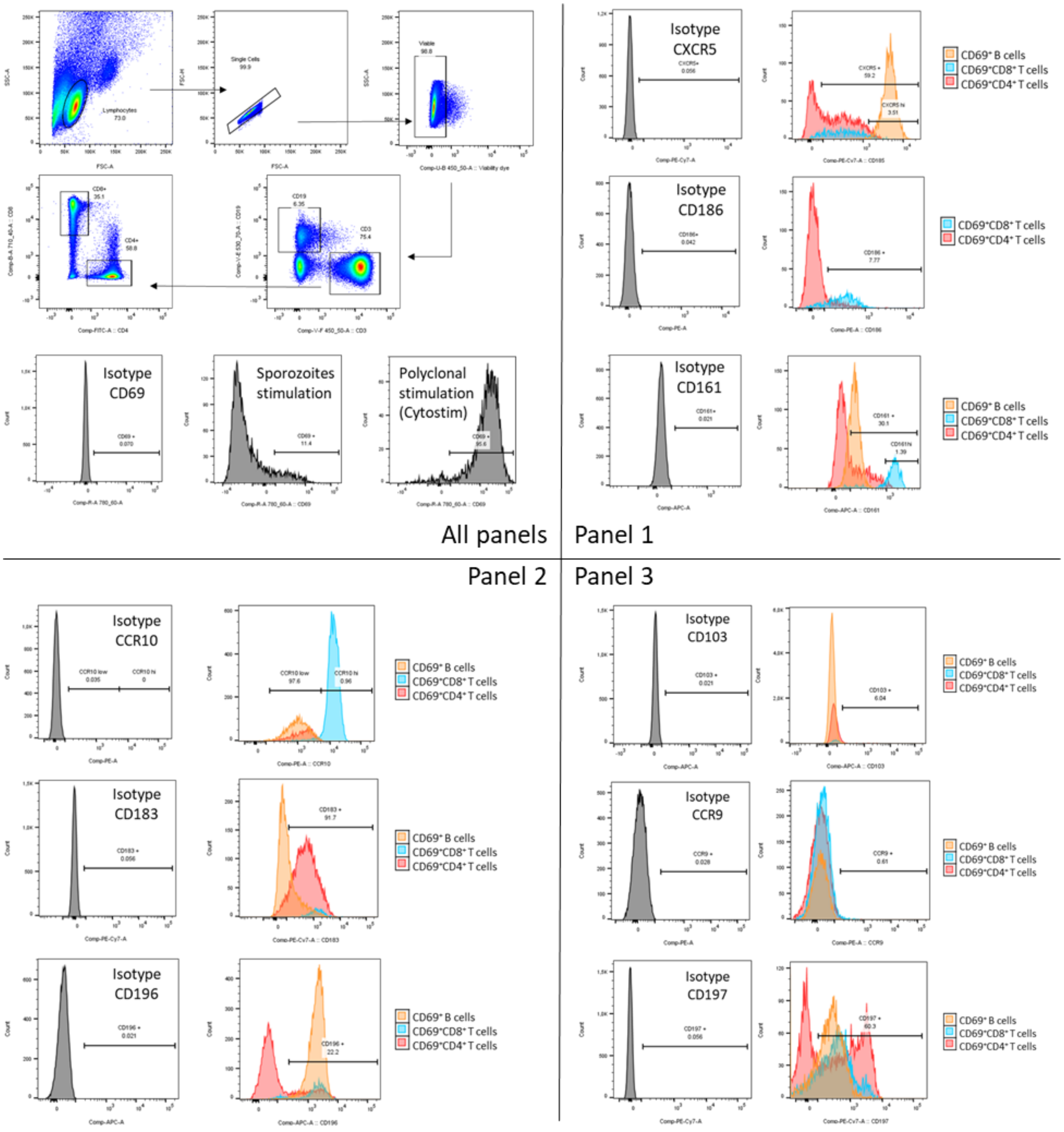

Supplementary Figure S2

Homing receptor profiles induced by RAS vaccination. The relationship between expressed homing receptors on SPZ-specific lymphocytes was investigated by generating a correlation matrix including all 16 parameters (lineage markers, homing receptors) after vaccination (T1, Panel A) and early after CHMI (T2, Panel B). The heatmap groups correlated immune measures by hierarchical clustering. The color indicates the degree of correlation between the different parameters (positive correlation in blue, negative correlation in red, highly correlated values in dark colors to weakly correlated values in light colors).

A

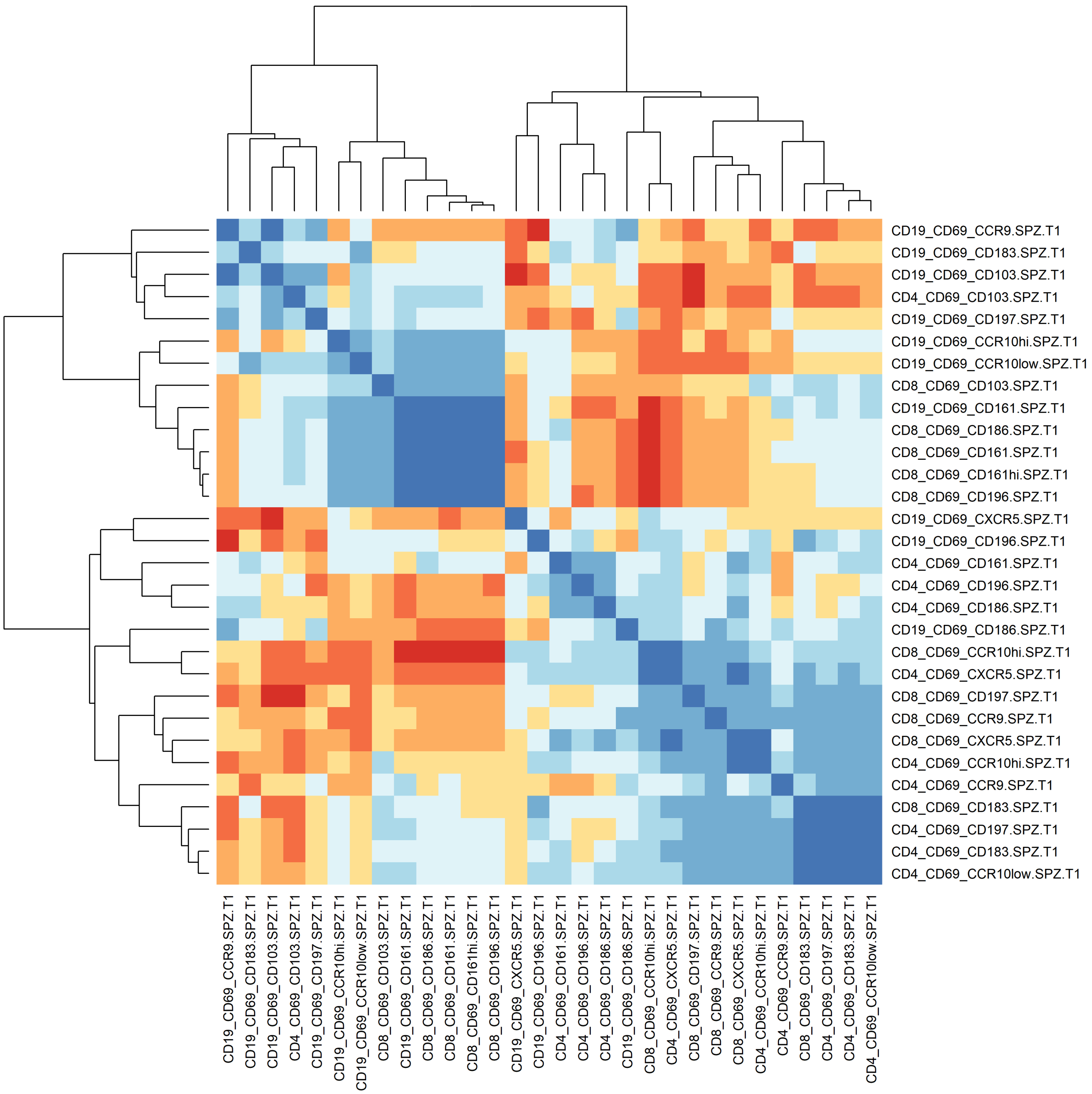

B

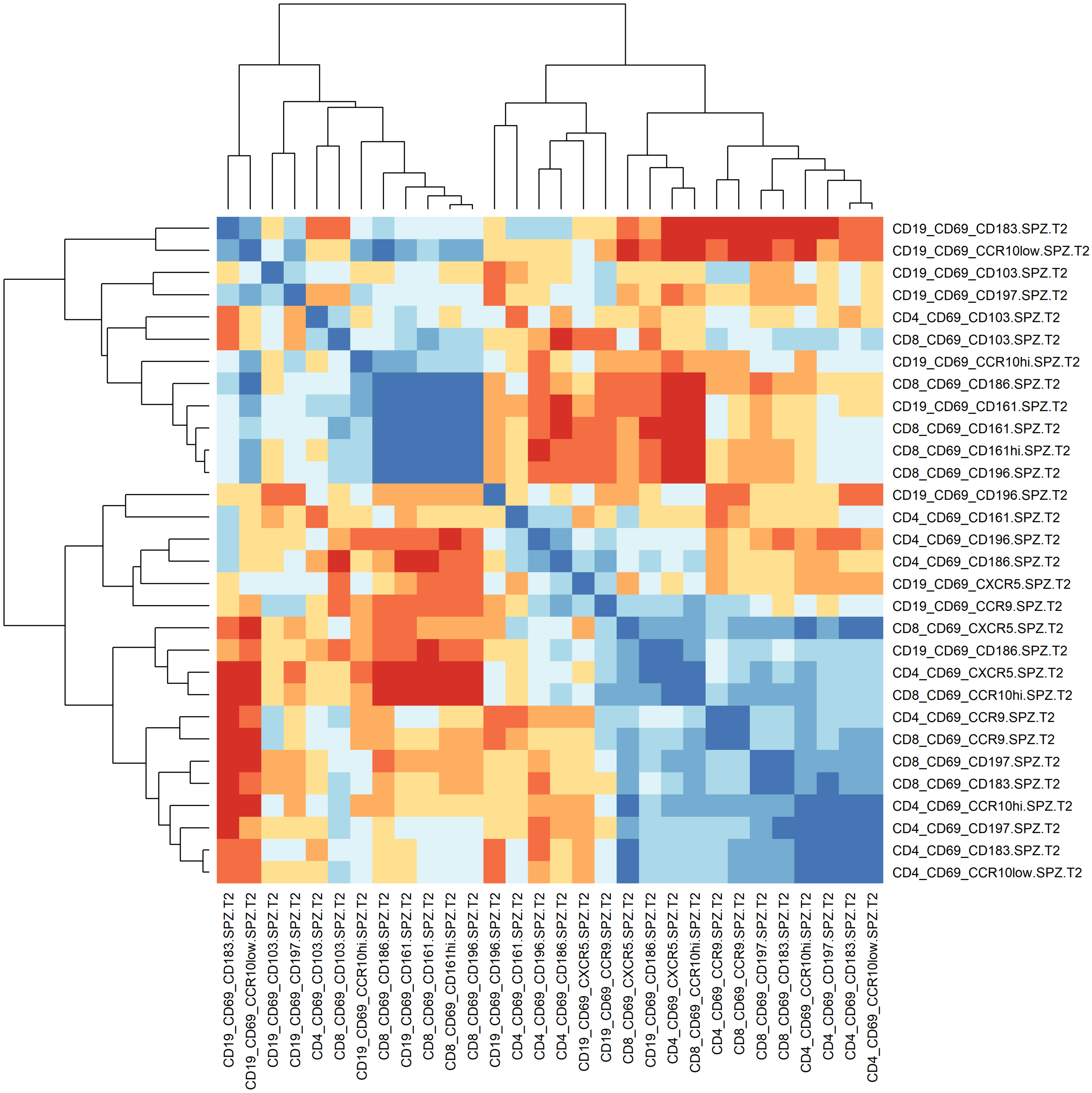

CD19_CD69_CD183.SPZ.T2
CD19_CD69_CCR10low.SPZ.T2
CD19_CD69_CD103.SPZ.T2
CD19_CD69_CD197.SPZ.T2
CD4_CD69_CD103.SPZ.T2
CD8_CD69_CD103.SPZ.T2
CD19_CD69_CCR10hi.SPZ.T2
CD8_CD69_CD186.SPZ.T2
CD19_CD69_CD161.SPZ.T2
CD8_CD69_CD161.SPZ.T2
CD8_CD69_CD161hi.SPZ.T2
CD8_CD69_CD196.SPZ.T2
CD19_CD69_CD196.SPZ.T2
CD4_CD69_CD161.SPZ.T2
CD4_CD69_CD196.SPZ.T2
CD4_CD69_CD186.SPZ.T2
CD19_CD69_CXCR5.SPZ.T2
CD19_CD69_CCR9.SPZ.T2
CD8_CD69_CXCR5.SPZ.T2
CD19_CD69_CD186.SPZ.T2
CD4_CD69_CXCR5.SPZ.T2
CD8_CD69_CCR10hi.SPZ.T2
CD4_CD69_CCR9.SPZ.T2
CD8_CD69_CCR9.SPZ.T2
CD8_CD69_CD197.SPZ.T2
CD8_CD69_CD183.SPZ.T2
CD4_CD69_CCR10hi.SPZ.T2
CD4_CD69_CD197.SPZ.T2
CD4_CD69_CD183.SPZ.T2
CD4_CD69_CCR10low.SPZ.T2
